# Volume assessment comparing femoral vein and inferior vena cava among chest pain patients presenting to the emergency department

**DOI:** 10.1186/s12245-024-00762-7

**Published:** 2024-11-28

**Authors:** Neeraja A Nair, Freston Marc Sirur, Vimal Krishnan S

**Affiliations:** Department of Emergency Medicine, Kasturba Medical College, Manipal, Manipal Academy of Higher Education (MAHE), Manipal, Udupi, Karnataka India

**Keywords:** POCUS (point of Care Ultrasound), Femoral vein Collapsibility Index, Volume assessment, Inferior Vena Cava, Volume responsiveness, Emergency Department.

## Abstract

**Background:**

Inferior vena cava (IVC) diameter measurement using ultrasound for volume status assessment has shown satisfactory results and is being adopted in Emergency and critical care settings. IVC diameter can vary depending on the cardiac function, respiratory efforts, intraabdominal pressure, and mechanical ventilation. Due to these factors, IVC measurement cannot be considered a stand-alone technique appropriate for every patient. The femoral vein (FV), a more superficial vein than IVC, can be considered an alternative method for assessing fluid responsiveness in patients presenting to the Emergency department. It is easily accessible and can be used in scenarios where IVC cannot be visualized or reliable.

**Methods:**

This was a single-center diagnostic study where 85 patients who presented to the ED with chest pain were enrolled prospectively. IVC and femoral vein collapsibility indices, stroke volume, and cardiac output are measured using an ultrasound machine. The measurements were repeated after a passive leg-raising test. These values were compared with each other to assess an intra-class correlation between IVC and femoral vein collapsibility indices. We have also evaluated the relationship between the collapsibility indices of both veins and cardiac output.

**Discussion & limitations:**

Our findings show an insufficient correlation between IVC and FV collapsibility indices. However, both vein diameters significantly increased after passive leg raising (PLR), indicating a response to fluid challenge. Post-PLR reduced IVC, and FV collapsibility index (CI) suggests intravascular volume expansion after a fluid challenge, also reflected in the hemodynamic parameters. Our study was conducted only in a subset of relatively stable patients. The applicability of the study in different subsets of patients presenting to ED is still questionable.

**Conclusion:**

We conclude that femoral vein indices may not be an accurate alternative for volume assessment in the chosen cohort of patients. IVC and FV metrics do not correlate and may not be accurate for volume responsiveness. We may need to explore the utility of FV and its indices in a larger population in multiple settings for a better understanding of its role in volume assessment and responsiveness.

**Trial registration:**

(EC/NEW/INST/2021/1707). Registered 03 January 2023.

## Background

Volume resuscitation is a cornerstone of treating a critically ill patient presenting to the emergency department. Administering intravenous fluids can replenish intravascular volume, thereby enhancing stroke volume and cardiac output [1]. Resuscitation with excessive amount of fluid can lead to endothelial injury, leading to capillary leak, and interstitial edema with pulmonary and cardiac overload, which in turn leads to multi-organ dysfunction [2, 3].

Volume assessment is critical to patient care in various clinical settings, particularly in conditions such as shock, sepsis, heart failure, and fluid overload. It involves evaluating the patient’s fluid status to determine the appropriate fluid management strategy. In the current clinical practice, two broad categories of measures are used: Static and Dynamic [4]. While both static and dynamic measures play a role in fluid assessment, dynamic measures offer several advantages in clinical practice compared to static measures [5]. Static parameters are weak predictors of fluid responsiveness, proven through multiple studies over the past decade [6–8].

Volume assessment using point-of-care ultrasound evaluation of Inferior vena cava (IVC) diameter is a popular strategy adopted in Emergency and critical care settings but has its limitations [9]. There are, however, multiple confounders like cardiac function, respiratory efforts, intraabdominal pressure, and mechanical ventilation influencing assessment [10]. Due to these factors, IVC measurement cannot be considered a stand-alone technique appropriate for every patient. Integrating passive leg raising (PLR) test and cardiac output to assess volume responsiveness has been studied earlier with positive results [11, 12].

The femoral vein, more superficial than IVC, is theoretically an interesting alternative site for volume assessment in emergency settings. The presumed benefits of easily accessible anatomical site and fewer barriers to visualization make it an attractive option for clinicians. It might overcome some of the technical confounders associated with IVC assessment [13, 14]. The femoral vein collapsibility index (FVCI) is calculated using ultrasound imaging, offering a non-invasive and rapidly deployable method to evaluate fluid responsiveness. The FVCI measures the degree of collapsibility of the femoral vein in response to changes in intrathoracic pressure, which can be induced by respiration or other maneuvers. This is an emerging tool for assessing a patient’s volume status, particularly in critical care and emergency medicine settings [15]. FV and IVC assessment for fluid responsiveness has been evaluated in a systematic review meta-analysis done by Kim et al. [20], which concluded that there are limited studies on other veins, including the femoral vein, for volume assessment. There is a need to explore the utility of the femoral vein collapsibility index (FVCI) in emergency settings.

We have integrated ultrasound-based femoral vein diameter assessment and passive leg raising (PLR) test in our study to find the utility of the femoral vein in predicting volume responsiveness in patients. We aimed to evaluate the utility of FVCI and compare it with the conventional IVC assessment for chest patients presenting to the Emergency Department.

## Methods

### Study design & setting

This was a single-center experimental study conducted on patients with chest pain presenting to the Emergency Medicine Department of a tertiary care teaching hospital in South India after receiving IEC clearance and CTRI registration.

### Study population

The study population includes patients from the southern state of India, Karnataka.

### Sample size

To detect an effect size of 0.4 using a paired t-test at a 5% level of significance with 90% power, we require a minimum of 68 pairs. Since the outcome variable is not normally distributed, a non-parametric test must be performed, which requires the minimum sample size to be adjusted as 68 multiplied by 1.2. Thus, a hypothesized sample size of 82 is needed for comparison. Hence, 85 adults who presented to the Emergency Medicine Department with chest pain and consented to participate were enrolled after meeting the inclusion and exclusion criteria.

### Statistical methods

We used a paired t-test to compare the normally distributed data, and the Wilcoxon paired t-test was used for the rest.

### Inclusion criteria

Patients presented complaints of chest pain to the Emergency medicine department.

### Exclusion criteria

1. Patients with abdominal mass or other pathology.
2. Severe pulmonary artery hypertension.
3. Femoral vein occlusion.
4. Deep vein thrombosis, Inferior vena cava filter.
5. Pregnancy.
6. ST elevation MI (STEMI).
7. Pulmonary embolism.
8. Pulmonary oedema.
9. Heart failure with reduced EF.
10. Hemodynamic instability (Hypotension/ arrhythmia).
11. Mechanical ventilation.

The images are obtained using a single machine, the GE Versana^®^ active TM ultrasound machine, and captured by a single operator.

**Fig. 1:**
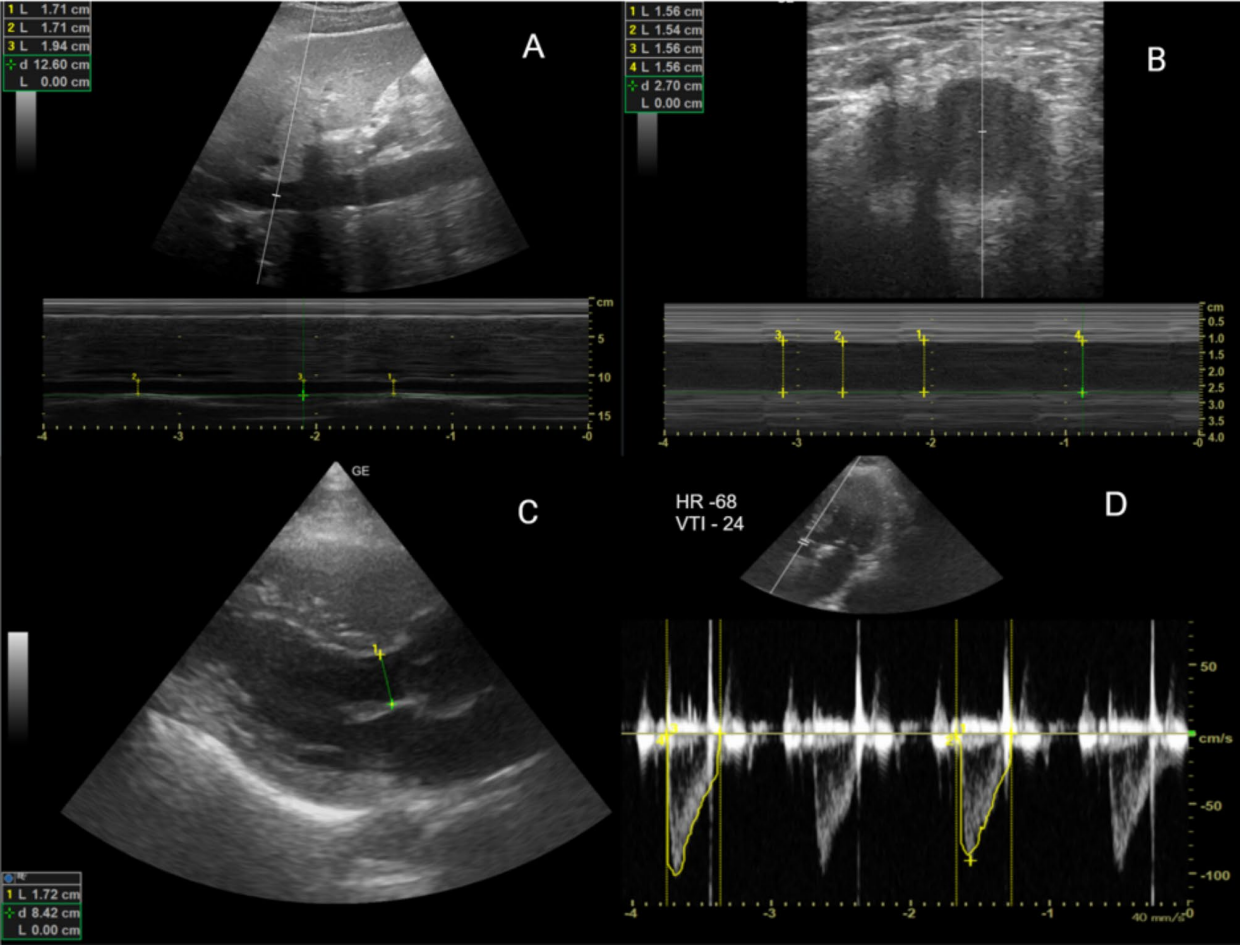
Ultrasound images of IVC, femoral vein & LVOT-VTI Image A -Ultrasound image of IVC using M mode. L1, L2, L3– 3 values of IVC diameters; d – depth at which IVC is measured Image B - Femoral artery(left), Femoral vein(right) L1, L2, L3, L4 – Diameters of femoral vein on inspiration and expiration; d – depth at which femoral vein is measured Image C - Ultrasound image of parasternal long axis view of LVOT; L – diameter of LVOT; d – depth at which LVOT is measured Image D - Ultrasound image of LVOT with VTI measurement in apical 5 chamber view VTI Velocity time integral of 24; HR – Heart rate of 68 bpm

After obtaining informed consent and explaining the procedure, the patient was placed in a supine position. Heart rate, blood pressure, oxygen saturation, and respiratory rate were monitored continuously during the procedure. Using a curvilinear probe(2-5Mhz) of the ultrasound placed in the subcostal area, a long-axis view of the inferior vena cava (IVC) was obtained and confirmed by visualizing the IVC entering the right atrium and a segment of the hepatic vein joining the IVC. After acquiring a good view of IVC, M-mode was used to obtain the respiratory phasic variation of IVC. The M-mode pointer was placed 2 cm away from the junction of the hepatic vein joining IVC. The image was frozen, and using calipers, maximum and minimum IVC diameters perpendicular to the long axis were measured (Fig. 1). IVC collapsibility index (IVC CI) was calculated using the formula.

IVC CI = (Maximum IVC diameter – Minimum IVC diameter) ÷ Maximum IVC diameter × 100.

**Technique**- With the linear probe(5-10Mhz), the right common femoral vein was identified using the inguinal ligament crease as the landmark, 2–4 cm below the level of the inguinal ligament, above the inguinal canal. The great saphenous vein take-off was traced by scanning caudally at the anteromedial aspect of the common femoral vein. The measurements were taken when the great saphenous vein was no longer seen caudally. DVT screening of the common femoral vein was also performed simultaneously. Using the M-mode of the ultrasound, the largest diameter of the vein is measured. Maximum and minimum FV diameter is measured with respiratory phasic variation (Fig. 1). The following formula calculates the femoral vein collapsibility index (FV CI);

FV CI = (Maximum FV diameter – Minimum FV diameter) ÷ Maximum FV diameter × 100.

A phased array transducer(1-2Mhz) was used to measure the stroke volume using the LVOT-VTI method. LVOT diameter was measured in parasternal long-axis view within 0.5 to 1 cm of the aortic annulus, as this location best reflects the exact anatomic location of the laminar LVOT velocity profile [16]. VTI measurements were taken in an apical 5-chamber view (Fig. 1). LVOT VTI was calculated by placing the pulse wave doppler in the outflow tract below the aortic valve and recording the velocity (cm/s). With the assumption of laminar flow through the LVOT, this measurement correlates well with cardiac output, which is the product of stroke volume and heart rate [17]. After attaining both values, stroke volume was calculated using the formula.

SV = (LVOT Diameter ÷ 2)^2^ × π × VTI.

A passive leg-raising (PLR) maneuver was done to assess both the femoral vein and IVC response to volume. From the supine position, the patient was shifted to a semi-recumbent position where the trunk was at 45°. Then, the patient’s upper body was lowered to a horizontal position while the lower limbs were elevated to 45°. All these positional changes were done by adjusting the bed without manipulating the patient. An angle of 45° was measured using a goniometer, and the bed was adjusted accordingly. After the passive leg raising test, all measurements were obtained within 2 min. PLR was repeated to obtain the values within the time limit of 2 min. Along with these, hemodynamic parameters mentioned in Table 1 are also monitored and documented during the maneuver.

A total of 2 sets of values, pre-PLR and post-PLR, of IVC diameter, femoral vein diameter, and stroke volume were measured and documented.

## Results

**Table 1:** Demographic characteristics

Variables (*N* = 85)	Mean	Standard deviation
Age	54.96	13.73
**Gender**	**Number**	**Percentage**
Male	50	58.80%
Female	35	41.20%

Table 1 summarizes a sample population’s demographic and clinical characteristics (*N* = 85). The mean age of the participants is 54.96 years, with a standard deviation of 13.73 years, indicating a moderate spread around the mean age. Gender distribution shows a higher proportion of males (58.8%) compared to females (41.2%).

**Table 2:** Comparison of pre and post-PLR parameters

Variables	Pre-PLR		Post-PLR		*p* value	Statistical test used
	Median	IQR	Median	IQR		
HR (bpm)	74	64–85	76	65–86	< 0.001	Wilcoxon signed rank test
RR (mnt)	18	16–20	18	17–20	0.747	Wilcoxon signed rank test
SBP (mmHg)	140	120–140	140	130–150	< 0.001	Wilcoxon signed rank test
DBP (mmHg)	80	80–90	84	80–90	0.004	Wilcoxon signed rank test
SPO2(%)	99	98–100	99	98–100	0.877	Wilcoxon signed rank test
IVC Max diameter(cm)	1.64	1.4–1.8	1.82	1.6–2.1	< 0.001	Wilcoxon signed rank test
IVC Min diameter(cm)	1.2	1-1.4	1.5	1.32–1.8	< 0.001	Wilcoxon signed rank test
IVC.CI	0.23	0.13–0.31	0.12	0.06–0.23	< 0.001	Wilcoxon signed rank test
FV Max diameter(cm)	1.11	1-1.3	1.3	1.13–1.5	< 0.001	Wilcoxon signed rank test
FV Min diameter(cm)	1.06	0.95–1.3	1.2	1.1–1.4	< 0.001	Wilcoxon signed rank test
FV.CI	0.05	0.03–0.08	0.04	0.02–0.06	< 0.001	Wilcoxon signed rank test
VTI	21.1	3.56	23	3.58	< 0.001	Paired t test
Stroke volume(mL/beat)	55.6	16	60	17	< 0.001	Paired t test
Cardiac output(L/mint)	4.1	13	4.5	14	< 0.001	Paired t test

HR - Heart rate; RR – Respiratory rate; SBP – Systolic blood pressure; DBP – Diastolic blood pressure; SpO2 – Peripheral oxygen saturation; IVC CI – Inferior vena cava collapsibility index; FV CI – Femoral vein collapsibility index; VTI – Velocity time integral

Table 2 compares pre-PLR and post-PLR hemodynamic parameters and IVC, femoral vein indices, cardiac output, and their significance with respect to volume responsiveness. We used paired t-tests to assess the normally distributed data using mean and standard deviation. The Wilcoxon t-test was used for the rest of the data using the median and interquartile range. Our results indicate a significant increase in IVC and femoral vein diameters and collapsibility indices post-PLR test. It also conveys that stroke volume and cardiac output significantly increase post-PLR.

**Fig. 2:**
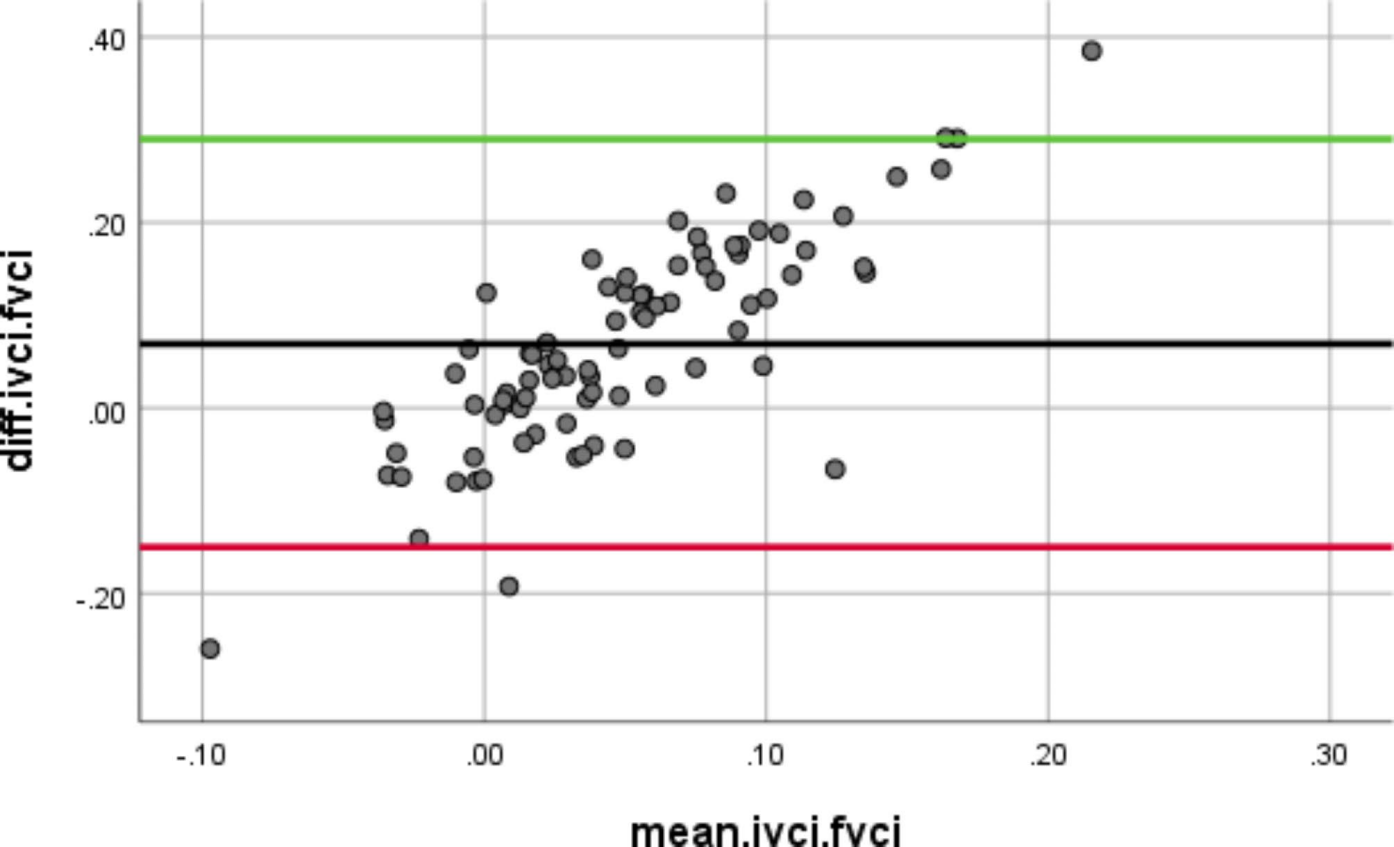
Bland-Altman plot X-Axis (mean.ivci.fvci); Y-Axis (diff.ivci.fvci)

Figure 2 represents the Bland-Altman plot, with the x-axis showing the mean IVC CI and FV CI and the y-axis showing the difference between IVC CI and FV CI.

**Table 3:** Comparison of IVC and FV collapsibility indices with cardiac output

Variables	Correlation coefficient (ρ)	*P* value
**Pre-PLR**		
IV CI v/s Cardiac output	-0.042	0.704
FV CI v/s Cardiac output	0.058	0.601
**Post-PLR**		
IV CI v/s Cardiac output	-0.042	0.704
FV CI v/s Cardiac output	0.030	0.787

IVC CI - Inferior vena cava collapsibility index; FV CI – Femoral vein collapsibility index

Table 3 presents the results of Spearman bivariate correlations between collapsibility indices (IV CI and FV CI) and cardiac output (CO) both before and after PLR. The results suggest that neither IV CI nor FV CI significantly correlates with cardiac output, both before and after PLR. This implies that changes in IVC and FV collapsibility indices may not predict changes in cardiac output in this context.

## Discussion

Our findings indicate an insufficient intraclass correlation between the IVC and FV collapsibility indices (Fig. 2). A substantial reduction in both IVC CI and FV CI was observed after PLR, suggestive of intravascular volume expansion occurring with redistribution of fluid into the central circulation. These changes are reflected in heart rate and systolic blood pressure, which appear to be elevated post-PLR test (Table 2).

We compared both IVC and FV collapsibility indices with stroke volume and cardiac output using point-of-care echo before and after PLR in all participants (Table 3). We found only a weak correlation between cardiac output and both collapsibility indices. Our results show that IVC and FV are not accurate volume assessment methods in this population compared to cardiac output measurement.

Our results are synchronous with those of Kent et al. [18] study, showing only a weak correlation between FV-CI and IVC-CI.

However, the subsets of patients Kent et al. compared had both mechanically ventilated and spontaneously breathing patients. The outcomes could be significantly impacted by such a diverse population with varying physiologies. Our study had a homogenous population of spontaneously breathing patients where venous physiology is not altered by mechanical ventilation.

Study of Nedel et al. [19] on the respiratory variation of femoral vein diameter in mechanically ventilated patients concluded that femoral vein collapsibility has moderate accuracy for fluid responsiveness in septic shock compared to IVC, which showed a greater accuracy. The methodology of this study is closer to ours as they have performed a PLR test followed by cardiac output to measure fluid responsiveness. The study population we chose may have contributed to variations in our results. While we conducted the study in a group of spontaneously breathing and hemodynamically stable patients, Nedel et al. chose a population of critically ill patients on mechanical ventilation.

Studies by Cho et al. [20], and Begum et al. [21], Malik et al. [22] consistently show that femoral vein diameter (FVD) correlates with central venous pressure (CVP).

Compared to other studies, we have assessed the IVC and FV diameters before and after the PLR test, which gives us a general understanding of how these diameters change with volume expansion. We also computed collapsibility indices for IVC and femoral veins before and after the PLR test. This maneuver allows physicians to evaluate changes in both veins without resorting to an actual fluid challenge. We have considered LVOT VTI as a standard method to assess volume responsiveness non-invasively and calculated SV to ensure objectivity for comparison. Most of the studies in our analysis did not compare their findings to an institutional standard approach.

Femoral vein assessment was explored as an option in the Emergency Department due to its easy accessibility, as it is more exposed and superficial than IVC. However, the femoral vein can get easily compressed due to external factors, especially during sonographic assessment.

Consequently, a significant variation in the reported collapsibility indices may be explained by even little variations in the pressure applied to the ultrasonic probe during the acquisition of both FV measurements. We were cognizant of this possible confounder and used a consistent recording technique without significantly altering the venous geometry.

We have observed that respiratory phasic variation has a bigger impact on IVC diameter than FV diameter. This variation might be due to the proximity of IVC to the diaphragm, which impacts the venous system. This may have affected the study’s collapsibility index (CI) metrics and influenced the intraclass correlation. Our study results indicate that IVC and femoral vein might not be comparable because of their differences in anatomical location, vessel wall properties, and confounding factors such as respiration, abdominal pressures, etc. In the present study, the femoral vein assessment did not correlate with IVC assessment or reflect the accuracy of echo-guided indices in volume assessment.

### Limitations

Although we tried to ensure homogeneity with the selected population, the smaller sample size and the fact that the study was carried out in a single center are the limitations we acknowledge.

We have conducted the study only in one subset of relatively stable patients. The applicability of the study in different subsets of patients presenting to ED is still questionable.

A complete DVT scan is not performed in all patients, although it was an exclusion criterion of the study.

## Conclusion

We conclude that Femoral vein indices may not be an accurate alternative for volume assessment in the chosen cohort of patients. IVC and FV metrics do not correlate and may not be accurate for volume responsiveness. We may need to explore the utility of FV and its indices in a larger population in multiple settings for a better understanding of its role in volume assessment and responsiveness.

## Data Availability

No datasets were generated or analysed during the current study.

## Abbreviations

IVC: Inferior vena cava
IVC CI: Inferior vena cava collapsibility index
FV: Femoral vein
FV CI: Femoral vein collapsibility index
CVP: Central venous pressure
PCWP: Pulmonary capillary wedge pressure
LVOT: Left ventricular outflow tract
VTI: Velocity time integral
PLR: Passive leg raising
PPV: Pulse pressure variability
SPV: Systolic pressure variability
SVV: Stroke volume variability
POCUS: Point of care ultrasound
SV: Stroke volume
CO: Cardiac output
IJV: Internal jugular vein
SVC: Subclavian vein
HR: Heart rate
RR: Respiratory rate
BP: Blood pressure
SBP: Systolic blood pressure
DBP: Diastolic blood pressure
SpO2: Saturation of peripheral oxygen
SD: Standard deviation
AUROC: Area under the curve
ED: Emergency department
DVT: Deep vein thrombosis

## References

[1] Cherpanath TGV, Geerts BF, Lagrand WK, Schultz MJ, Groeneveld ABJ. Basic concepts of fluid responsiveness. Neth Heart J. 2013;21(12):530–6. 24170232 10.1007/s12471-013-0487-7 PMC3833913

[2] Malbrain MLNG, Marik PE, Witters I, Cordemans C, Kirkpatrick AW, Roberts DJ, et al. Fluid overload, de-resuscitation, and outcomes in critically ill or injured patients: a systematic review with suggestions for clinical practice. Anaesthesiol Intensive Ther. 2014;46(5):361–80. 25432556 10.5603/AIT.2014.0060

[3] Huang ACC, Lee TYT, Ko MC, Huang CH, Wang TY, Lin TY, et al. Fluid balance correlates with clinical course of multiple organ dysfunction syndrome and mortality in patients with septic shock. PLoS ONE. 2019;14(12):e0225423. 31790451 10.1371/journal.pone.0225423 PMC6886786

[4] Mackenzie DC, Noble VE. Assessing volume status and fluid responsiveness in the emergency department. Clin Exp Emerg Med. 2014;1(2):67–77. 27752556 10.15441/ceem.14.040 PMC5052829

[5] Guerin L, Monnet X, Teboul JL. Monitoring volume and fluid responsiveness: from static to dynamic indicators. Best Pract Res Clin Anaesthesiol. 2013;27(2):177–85. 24012230 10.1016/j.bpa.2013.06.002

[6] Monnet X, Marik PE, Teboul JL. Prediction of fluid responsiveness: an update. Ann Intensive Care. 2016;6:111. 27858374 10.1186/s13613-016-0216-7 PMC5114218

[7] Jalil BA, Cavallazzi R. Predicting fluid responsiveness: a review of literature and a guide for the clinician. Am J Emerg Med. 2018;36(11):2093–102. 30122506 10.1016/j.ajem.2018.08.037

[8] Weigl W, Adamski J, Onichimowski D, Nowakowski P, Wagner B. Methods of assessing fluid responsiveness in septic shock patients: a narrative review. Anaesthesiol Intensive Ther. 2022;54(2):175–83. 35413788 10.5114/ait.2022.115368 PMC10156515

[9] Long E, Oakley E, Duke T, Babl FE, Paediatric Research in Emergency Departments International Collaborative (PREDICT). Does respiratory variation in Inferior Vena Cava Diameter Predict Fluid responsiveness: a systematic review and Meta-analysis. Shock Augusta Ga. 2017;47(5):550–9. 28410544 10.1097/SHK.0000000000000801

[10] Kaptein MJ, Kaptein EM. Inferior Vena Cava Collapsibility Index: clinical validation and application for Assessment of relative intravascular volume. Adv Chronic Kidney Dis. 2021;28(3):218–26. 34906306 10.1053/j.ackd.2021.02.003

[11] Cherpanath TGV, Hirsch A, Geerts BF, Lagrand WK, Leeflang MM, Schultz MJ, et al. Predicting Fluid Responsiveness by Passive Leg raising: a systematic review and Meta-analysis of 23 clinical Trials*. Crit Care Med. 2016;44(5):981. 26741579 10.1097/CCM.0000000000001556

[12] Cavallaro F, Sandroni C, Marano C, La Torre G, Mannocci A, De Waure C, et al. Diagnostic accuracy of passive leg raising for prediction of fluid responsiveness in adults: systematic review and meta-analysis of clinical studies. Intensive Care Med. 2010;36(9):1475–83. 20502865 10.1007/s00134-010-1929-y

[13] Via G, Tavazzi G, Price S. Ten situations where inferior vena cava ultrasound may fail to accurately predict fluid responsiveness: a physiologically based point of view. Intensive Care Med. 2016;42(7):1164–7. 27107754 10.1007/s00134-016-4357-9

[14] Millington SJ. Ultrasound assessment of the inferior vena cava for fluid responsiveness: easy, fun, but unlikely to be helpful. Can J Anesth Can Anesth. 2019;66(6):633–8. 10.1007/s12630-019-01357-0 30919234

[15] Kim DW, Chung S, Kang WS, Kim J. Diagnostic accuracy of Ultrasonographic Respiratory Variation in the Inferior Vena Cava, Subclavian Vein, Internal Jugular Vein, and femoral vein diameter to Predict Fluid responsiveness: a systematic review and Meta-analysis. Diagn Basel Switz. 2021;12(1):49. 10.3390/diagnostics12010049 PMC8774961 35054215

[16] Hahn RT, Pibarot P. Accurate Measurement of Left Ventricular Outflow Tract Diameter: comment on the updated recommendations for the Echocardiographic Assessment of Aortic Valve Stenosis. J Am Soc Echocardiogr. 2017;30(10):1038–41. 28764864 10.1016/j.echo.2017.06.002

[17] Aligholizadeh E, Teeter W, Patel R, Hu P, Fatima S, Yang S, et al. A novel method of calculating stroke volume using point-of-care echocardiography. Cardiovasc Ultrasound. 2020;18(1):37. 32819371 10.1186/s12947-020-00219-w PMC7441555

[18] Kent A, Patil P, Davila V, Bailey JK, Jones C, Evans DC, et al. Sonographic evaluation of intravascular volume status: can internal jugular or femoral vein collapsibility be used in the absence of IVC visualization? Ann Thorac Med. 2015;10(1):44–9. 25593607 10.4103/1817-1737.146872 PMC4286845

[19] Nedel WL, Simas DM, Marin LG, Morais VD, Friedman G. Respiratory variation in femoral vein diameter has moderate accuracy as a marker of fluid responsivity in mechanically ventilated septic shock patients. Ultrasound Med Biol. 2017;43(11):2713–7. 28756901 10.1016/j.ultrasmedbio.2017.06.023

[20] Cho RJ, Williams DR, Leatherman JW. Measurement of femoral vein diameter by Ultrasound to Estimate Central venous pressure. Ann Am Thorac Soc. 2016;13(1):81–5. 26561731 10.1513/AnnalsATS.201506-337BC

[21] Begum M, Arshad AR, Hussain A, Khalid A. Is femoral vein Diameter a Reliable marker of central venous pressure? Life Sci. 2023;4:132–5.

[22] Malik A, Akhtar A, Saadat S, Mansoor S. Predicting Central venous pressure by measuring femoral venous diameter using Ultrasonography. Cureus. 2016;8(11):e893. 28018763 10.7759/cureus.893 PMC5178981

